# Risk factors for neonatal bronchopulmonary dysplasia in extremely preterm premature rupture of membranes: a retrospective study

**DOI:** 10.1186/s12884-020-03366-0

**Published:** 2020-11-03

**Authors:** Eishin Nakamura, Shigetaka Matsunaga, Yoshihisa Ono, Yasushi Takai, Hiroyuki Seki

**Affiliations:** 1 Center for Maternal, Fetal and Neonatal Medicine, Saitama Medical Center, Saitama Medical University, 1981 Kamoda, Kawagoe-shi, 350-8550 Saitama, Japan; 2 Department of Gynecology, Saitama Medical Center, Saitama Medical University, Saitama, Japan

**Keywords:** preterm premature rapture of membrane, preterm labour, oligohydramnios, bronchopulmonary dysplasia

## Abstract

**Background:**

Determination of the optimal timing for termination of pregnancy in cases of preterm premature rupture of membranes (pPROM) during the extremely preterm period is still difficult. Bronchopulmonary dysplasia (BPD) is a major disease widely taken into account when determining the prognosis of respiratory disorders in a neonate. Many aspects of this disease remain unclear. With the aim of further improving the prognosis of neonates born to mothers with pPROM, this study examined cases who were diagnosed with pPROM before 28 weeks of gestation. The study analysed risk factors for neonatal BPD.

**Methods:**

This study included 73 subjects with singleton pregnancy, diagnosed with pPROM during the gestational period from 22 weeks and 0 days to 27 weeks and 6 days. The following factors were retrospectively examined: the gestational week at which pPROM was diagnosed, the gestational week at which delivery occurred, the period for which the volume of amniotic fluid was maintained, and neonatal BPD as a complication. Receiver operating characteristic (ROC) curve analyses were conducted to analyse the relationship of the onset of BPD with the duration of oligohydramnios and the gestational weeks of delivery.

**Results:**

The mean gestational week at which a diagnosis of amniorrhexis was made was 24.5 ± 1.9 weeks (mean ± SD), and that at which delivery occurred was 27.0 ± 3.0 weeks. Fifty-seven cases (78.1%) were diagnosed with oligohydramnios, the mean duration of which was 17.4 ± 20.5 days. The mean birth weight of neonates was 1000 ± 455 g, of which 49 (67.1%) were diagnosed with BPD following birth. No neonates died in this study. The ROC curve indicated that the cut-off values for the duration of oligohydramnios and gestational age at delivery were 4 days and 24.1 weeks, respectively. Multivariate analysis indicated that the duration of oligohydramnios for more than 4 days before delivery and preterm delivery at less than 24.1 weeks were risk factors for the onset of BPD.

**Conclusions:**

Our findings suggest that duration of oligohydramnios for more than 4 days before delivery and preterm delivery less than 24.1 weeks are risk factors for BPD in cases who are diagnosed with pPROM before 28 weeks of gestation.

## Background

Premature rupture of membranes is associated with a high risk of chorioamnionitis (CAM) and is likely to cause oligohydramnios as a complication. This may lead to non-reassuring foetal status (NRFS) requiring early termination of pregnancy. However, in cases of preterm premature rupture of membranes (pPROM) during the extremely preterm period, it is difficult to determine the optimal timing for termination of pregnancy due to the following two opposing factors: foetal development that can be achieved by prolonging pregnancy; and damage to the foetus due to infection. To date, no consensus has been reached on this issue [1].

Meanwhile, a large number of cases in which neonates born to mothers with pPROM were complicated by bronchopulmonary dysplasia (BPD) have been reported [2]. Possible causes of neonatal BPD in pPROM cases include the underdevelopment of the lungs due to premature birth [3]; the impact of CAM [4], and a decrease in pulmonary extensibility accompanying oligohydramnios [5]. While it is considered that these causes are intricately interrelated, there is a dearth of research on predictors of BPD in cases diagnosed with pPROM during the extremely preterm period(less than 28 weeks of gestation).

This study retrospectively analysed the treatment results of cases who were diagnosed with pPROM before 28 weeks of gestation, and the prognosis of neonates following delivery, particularly the onset of BPD. The study aimed to elucidate predictors of BPD and accumulate primitive data that may contribute to clinical decision-making for cases who are diagnosed with pPROM before 28 weeks of gestation.

## Methods

This study retrospectively analysed 73 singleton pregnancy cases diagnosed with pPROM during the gestational period from 22 weeks and 0 days to 27 weeks and 6 days. These cases were selected from 4833 cases who underwent delivery at our hospital from April 2013 to March 2018. The target patient population consisted of pregnant women who had been checked at our hospital from the beginning of pregnancy, and patients who were transferred to our hospital because of pPROM or threatened premature delivery. From there, we limited the cases in which the maternal and neonatal course could be confirmed retrospectively.

The following patient characteristics were analysed: mother’s age, previous delivery, delivery method (vaginal delivery or caesarean section), the administration of betamethasone to the mother, the gestational week at which a diagnosis of pPROM was made, time of delivery, oligohydramnios, and the presence of clinical CAM as a complication. Also, the following factors were analysed for perinatal prognosis: neonatal weight, APGAR score, umbilical arterial blood pH, small for gestational age (SGA) as a complication, neonatal death, use of mechanical ventilation management for the neonates use of mechanical ventilation management, neonatal sepsis as a complication, CAM stage in pathologic examination of the placenta, and BPD as a complication. Expected date of confinement was determined from the last menstrual period and ultrasonography of the first trimester. The gestational age was estimated from the last menstrual period and crown-rump length in early pregnancy.

### Diagnosis definition

The diagnosis of pPROM was made through a combination of the following methods: checking leaked amniotic fluid in speculum examination; basic pH testing; and quantitative testing for amniotic proteins. Oligohydramnios was defined as an amniotic fluid index (AFI) of less than 5 cm on transabdominal ultrasonography. The duration of oligohydramnios was not considered from the actual time of rupture but from the time it was detected by ultrasonography at a medical institution.

Blanc’s classification [6] was adopted in staging CAM in the pathological examination of the placenta. The most severe stage in this classification is Stage III, which is defined as a case in which the presence of inflammatory cells in the amniotic membranes is observed in pathological examination.

Diagnostic criteria by the National Institute of Child Health and Human Development (NICHD) [7] were adopted in making a diagnosis of BPD. In other words, a diagnosis of BPD was made when the administration of oxygen at a fraction of inspiratory oxygen (FiO2) > 21 was required for a duration of 28 days or longer.

In these cases, preterm infants were delivered in the presence of a neonatologist. Depending on the respiratory condition of the newborn, intubation management was performed, and he was admitted to the neonatal intensive care unit and underwent artificial respiration management.

### pPROM treatment procedure

This section explains a treatment plan at our hospital for cases diagnosed with pPROM before 28 weeks of gestation. A blood test was performed, especially for white blood cell count, when a case was diagnosed with pPROM. If it was confirmed that the mother was not complicated by clinical CAM, details of which were given below, a total of two doses of 12 mg of betamethasone was administered to the mother every 24 hours. However, in cases where early termination was required and 48 hours follow-up with betamethasone was not possible, or those with obvious CAM in which patients had a risk of maternal pulmonary oedema due to betamethasone [8], the administration of betamethasone was omitted, but the surfactant was always administered after birth.

Ritodrine hydrochloride, magnesium sulphate or a combination of these were consecutively administered as a tocolytic agent. For antibiotic therapy, 6 g/day of ampicillin/sulbactam (ABPC/SBT) was administered for one week following the diagnosis of pPROM. The well-being of the foetus were evaluated on NST two to four times a day. The patterns of foetal heart rate were evaluated each time. The mother was required to rest in bed, and an indwelling urinary catheter was inserted. The mother was closely monitored for deep venous thrombosis if she was required to rest in bed for an extended period of time. Provided that pregnancy could be prolonged by approximately two weeks, the level of rest might be increased. In such cases, the mother was closely monitored for deep venous thrombosis. Foetal ultrasonography was performed weekly to evaluate the growth of the foetus; transabdominal ultrasonography was performed twice weekly to evaluate the amount of amniotic fluid.

The early delivery of the foetus was determined in the following cases: (1) diagnostic criteria for clinical CAM were met; or (2) a diagnosis of NRFS was made based on foetal heart rate monitoring. Criteria by Lencki and colleagues [9] was used when making a diagnosis of clinical chorioamnionitis. According to the criteria, a case was diagnosed with the condition if the mother had a temperature of 38 °C or higher and exhibits any of the following conditions: (1) a tachycardia at a rate ≥ 100 bpm; (2) a tender uterus; (3) odour from vaginal discharge/amniotic fluid; or (4) a white blood cell count ≥ 15,000/µL. Alternatively, a case was diagnosed with the condition if the mother exhibited all of the above four conditions while her body temperature was lower than 38 °C. While c ardiotocography (CTG) is the gold standard for determining the termination of pregnancy[10], it was uncertain in determining foetal well-being during the early weeks of pregnancy in this study. Therefore, we comprehensively determined the conditions for termination based on the changes in inflammatory reactions in blood tests and BPS in ultrasonography. However, in cases where the pregnancy continued beyond 34 weeks of gestation and pPROM was diagnosed once but if the amniotic fluid outflow was not observed, the amniotic fluid space was maintained and there were no signs of infection, our policy was to continue pregnancy and aim for full-term labour to the best of our efforts. In these cases, preterm infants were delivered in the presence of a neonatologist. Depending on the respiratory condition of the neonate, intubation management was performed, and the neonate was sent to the neonatal intensive care unit for mechanical ventilation management.

### Sample size

We estimated the sample size with reference to an existing report on the development of BPD and oligohydramnios[11, 12]. By assuming the incidence of BPD in patients with oligohydramnios as 0.4, α error as 0.05, and β error as 0.20, the required sample size was calculated to be 34.

### Statistical analysis

IBM SPSS Statistics for Windows, version 25® (IBM Corp., Armonk, N.Y., USA) was used for statistical analysis. Continuous variables were compared using the t-test whereas categorical variables were compared using the Fisher’s exact or χ2 test. Multiple logistic regression analysis was used for multivariate analysis. The following variables were considered confounding factors: gestational age at delivery, duration of oligohydramnios, CAM stage III in pathologic examination of the placenta, small for gestational age, the male sex of the neonate, and the use of positive pressure ventilation after birth. The level of statistical significance was set at p < 0.05. The cut-off value for the receiver operating characteristic (ROC) curve was set by using Youden’s index [13]. In this method, the cut-off point is defined as the point where the sum of the levels of sensitivity and specificity[14] reaches its maximum on the ROC curve. At our hospital, termination of pregnancy is determined when a case is diagnosed with clinical CAM. Hence, 16 cases who were diagnose with clinical CAM during pregnancy were excluded from analysis in which the ROC curve was used. In the ROC curve, the remaining 57 cases were analysed.

In this study, we used multivariate analysis (multiple logistic analysis) including known confining factors such as preterm labor, duration of oligohydramnios, chorioamnionitis, small for gestational age, male sex of the neonate, and use of positive pressure ventilation after birth. It was analyzed after including it.

## Results

Table 1 shows patient background factors. The mean gestational week at which the case was diagnosed with premature rupture of membranes was 24.5 ± 1.9 weeks (mean ± standard deviation). Seventy-two patients (98.6%) gave premature birth at mean gestational week 27.0 ± 3.0. Oligohydramnios was observed in 57 patients (78.1%) with a mean duration of 17.4 ± 20.5 days, and anhydramnios was observed in 24 patients (32.9%). Sixteen patients (21.9%) were diagnosed with clinical CAM during pregnancy.

**Table 1:** Patient clinical characteristics.

	*n* = 73
Maternal age(years)	32.6 ± 5.2
Primiparity	31 (42.5%)
Delivery by caesarean section	56 (76.7%)
Administration of corticosteroid	59 (80.8%)
Gestational age at PROM (weeks)	24.5 ± 1.9
Gestational age at delivery (weeks)	27.0 ± 3.0
Delivery at less than 37 weeks of gestation	72 (98.6%)
≧34 weeks	2
30–33 weeks	8
26–29 weeks	31
22–25 weeks	31
Days form PROM to delivery (days)	17.4 ± 20.5
Clinical CAM	16 (21.9%)
^a^Oligohydramnios	57 (78.1%)
^b^Anhydramnios	24(32.9%)
Duration of oligohydramnios before delivery (days)	17.4 ± 20.5

^a^Oligohydramnios: Oligohydramnios was defined as an amniotic fluid index (AFI) of ≤ 5cm on transabdominal ultrasonography.

^b^Anhydramnios: Anhydramnios was defined as the complete lack of amniotic fluid on transabdominal ultrasonography.

Table 2 shows perinatal prognosis. The mean birth weight was 1000 ± 455 g, and small for gestational age (SGA) was observed in four cases (5.5%). No neonates died in this study. Forty-four cases (60.3%) were diagnosed with CAM stage III in postpartum pathologic examination of the placenta. Sixty-seven cases (91.8%) required positive pressure ventilation (PPV) following birth, of which six (8.2%) were diagnosed with neonatal sepsis. Forty-nine cases (67.1%) were diagnosed with neonatal BPD.

**Table 2:** Perinatal prognosis

	*n* = 73
Birth weight (g)	1000 ± 455
Male sex of the neonate	39 (53.4%)
Small for gestational age (< 10% tile)	4 (5.5%)
APGAR score (1 min)	4.4 ± 2.1
APGAR score (5 min)	6.6 ± 1.7
Umbilical artery pH	7.34 ± 0.08
Neonatal death	0
Use of positive pressure ventilation after birth	67 (91.8%)
^a^Neonatal sepsis	6 (8.2%)
CAM stage III in pathologic examination of the placenta	44 (60.3%)
Bronchopulmonary dysplasia	49 (67.1%)

Results are expressed as mean ± standard deviation or number (%)

^a^Neonatal death: defined as the death of the neonate within 28 days after birth

Table 3 shows the results of univariate analysis comparing the BPD and non-BPD groups. In univariate analysis, the number of gestational weeks at which rupture of membranes occurred was significantly smaller in the BPD group, but no significant difference was observed between the groups in terms of the number of gestational weeks at which delivery occurred. When cases were stratified by the number of gestational weeks at which delivery occurred, it was found that a significantly larger number of cases gave birth at gestational week 22–25 (odds ratio: 3.125, 95%CI: 1.083–8.945), which is the stratum for the earliest delivery, in the BPD group than in the non-BPD group. Also, a significantly larger number of cases were complicated by oligohydramnios in the BPD group (Odds ratio: 11.25, 95%CI: 3.185–39.27). The duration of the condition was significantly longer in this group as well. Apart from these, no significant differences were observed between the groups in aspects which have already been reported as risk factors for BPD, such as complication with SGA [15], the male sex of the neonate [16], and complication with chorioamnionitis [17].

**Table 3:** Results of univariate analysis for the risks of BPD

	With BPD (*n* = 49)	Without BPD (*n* = 24)	*P* value
Maternal age (years)	32.2 ± 5.4	33.4 ± 4.8	n.s.
Primiparity	18 (36.7%)	13(54.1%)	n.s.
Delivery by caesarean section	36(73.5%)	20 (83.3%)	n.s.
Administration of corticosteroid	41 (83.7%)	18 (75%)	n.s.
Gestational age at PROM (weeks)	23.9 ± 1.7	25.6 ± 1.7	< 0.05
Gestational age at delivery (weeks)	26.6 ± 2.9	27.6 ± 3.3	n.s.
Delivery at less than 37 weeks of gestation	49(100%)	23(95.8%)	n.s.
≧ 34weeks	1	1	n.s.
30-33weeks	5	3	n.s.
26-29weeks	18	13	n.s.
22-25weeks	26	6	< 0.05
Days form PROM to delivery (days)	18.9 ± 18.8	14.3 ± 23.6	n.s.
Clinical CAM	13 (26.5%)	3 (12.5%)	n.s.
^a^Oligohydramnios	45(91.8%)	12(50%)	0.05
Duration of oligohydramnios before delivery (days)	16.7 ± 18.3	2.2 ± 1.6	< 0.05
Birth weight (g)	968 ± 414	1065 ± 533	n.s.
Male sex of the neonate	27(55.1%)	12(50%)	n.s.
Small for gestational age (< 10% tile)	2(4.08%)	2(8.3%)	n.s.
Use of positive pressure ventilation after birth	46 (93.9%)	21(87.5%)	n.s.
Neonatal sepsis	4(8.16%)	2(8.3%)	n.s.
CAM stage III in pathologic examination of the placenta	30(61.2%)	14(58.3%)	n.s.

Results are expressed as mean ± standard deviation or number (%). Fisher’s extract exact test and t-test were used for two-group comparisons^a^Oligohydramnios was defined as an amniotic fluid index of ≤ 5 cm on transabdominal ultrasonography.

**Fig. 1:**
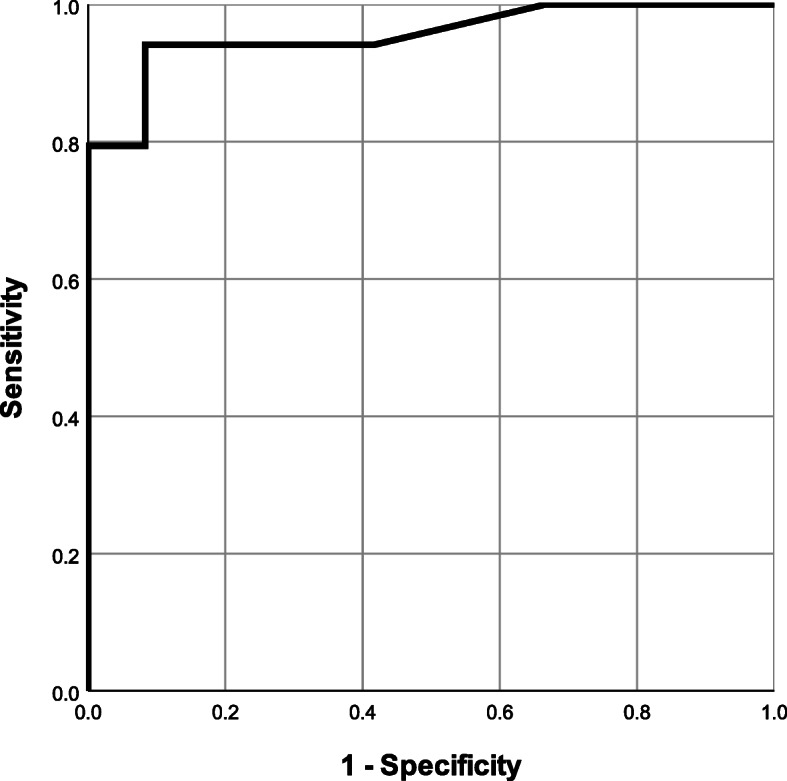
A receiver operating characteristic curve for the number of days for which oligohydramnios persisted in relation to the onset of neonatal BPD. The cut-off value was calculated by using Youden’s index. Sixteen cases who were diagnosed with clinical CAM during pregnancy were excluded from a total of 73 cases. Analysis was performed for the remaining 57 cases. AUC was 0.956 (95% CI: 0.902-1.000). The cut-off value for the duration of oligohydramnios as a risk factor for BPD was four days. In this case, the levels of sensitivity and specificity in predicting the onset of neonatal BPD were 0.941 and 0.917 respectively

Figure 1 shows an ROC curve for the duration of oligohydramnios (in days) in relation to the onset of neonatal BPD. The degree of correlation was analysed by using the curve. The area under the curve (AUC) of the duration of oligohydramnios (in days) relative to the onset of neonatal BPD was 0.956 (95% CI: 0.902-1.000). Using Youden’s index, the cut-off value for the duration of oligohydramnios (in days) as a risk factor for BPD was four days. In this case, the levels of sensitivity and specificity in predicting the onset of neonatal BPD were 0.941 and 0.917, respectively.

**Fig. 2:**
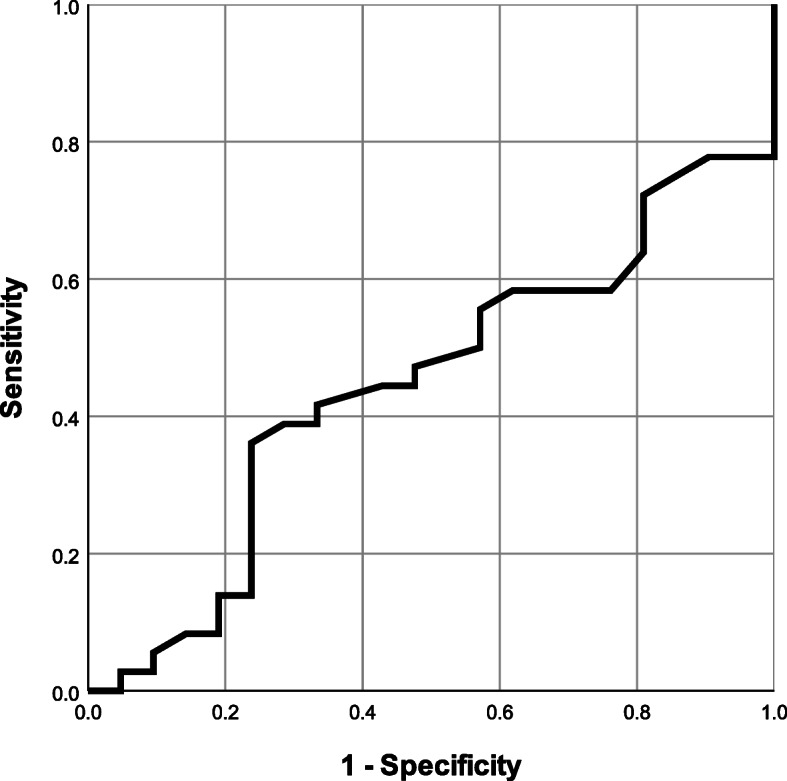
an ROC curve for the onset of BPD in neonates in relation to the gestational age at labour. The AUC was 0.555 (95%CI; 0.401–0.709), and the cut-off for the gestational age at risk of developing BPD was 24.1 weeks. The cut-off value could predict BPD development with a sensitivity of 0.222 and a specificity of 1.000

Figure 2 shows an ROC curve for the onset of BPD in neonates in relation to the gestational age at labour. The AUC was 0.555 (95%CI; 0.401–0.709), and the cut-off for the gestational age at risk of developing BPD was 24.1 weeks. The cut-off value could predict BPD development with a sensitivity of 0.222 and a specificity of 1.000.

The results of multivariate analysis for the onset of neonatal BPD are shown in Table 4.　The duration of oligohydramnios was divided into 2 groups (4 days or more, and less than 3 days) according to the cut-off value calculated in Fig. 1, and multivariate analysis was performed. Furthermore, the gestational age at delivery was also divided into 24.1 weeks or more and less than 24.1 weeks using the cut-off value calculated in Fig. 2. The results of multivariate analysis revealed that a duration of oligohydramnios of more than 4 days before delivery (odds ratio 6.240, 95%CI: 1.710–22.70) and gestational age less than 24.1 weeks at delivery (odds ratio 18.80, 95%CI: 1.160–306.0) were risk factors for BPD development.

**Table 4:** Results of multivariate analysis for the risks of BPD

	Odds ratio	95% CI	*P* value
Gestational age at delivery for less than 24.1 weeks	18.80	1.160–306.0	< 0.05
CAM stage III in pathologic examination of the placenta	0.888	0.274–2.880	n.s.
Male sex of the neonate	1.630	0.523–5.080	n.s.
Small for gestational age(< 10% tile)	0.127	0.009–1.880	n.s.
Duration of oligohydramnios for more than 4 days before delivery	6.240	1.710–22.70	< 0.05
Use of positive pressure ventilation after birth	7.870	0.749–82.60	n.s.

Multiple logistic analysis was used for multivariate analysis. *n.s.* not significant

## Discussion

In the present study, majority of the neonates (67.1%) delivered by the mothers suffering from pPROM were diagnosed with BPD. The univariate analysis showed that the gestational age at which membrane rupture occurred was significantly smaller in the BPD group and most of these cases were complicated by oligohydramnios. Furthermore, the multivariate analysis showed that the duration of oligohydramnios for more than 4 days before delivery and gestational age less than 24.1 weeks at delivery may be the risk factors for BPD. Additionally, analysis in which an ROC curve was used indicated that oligohydramnios that lasts for four days or longer may be a risk factor for BPD.

No consensus has been reached on how to deal with pPROM cases. At present, each case is individually treated, taking into consideration factors such as: estimated foetal weight, number of gestational weeks, the capacity of the facility to hundle neonatal resuscitation and management [18]. Past studies have found that prolonged oligohydramnios is associated with contracture and abnormalities of the musculoskeletal system [19] as well as pulmonary hypoplasia [20].

It is still extremely difficult to determine whether the termination of pregnancy is appropriate or not for pPROM cases. The following conditions may be clear criteria for the termination of pregnancy in pPROM cases: NRFS, clinical CAM, and clearly recognisable premature separation of the normally implanted placenta [1]. On the other hand, if these conditions are not observed, the termination of pregnancy must be carefully determined on an individual basis by taking into account the number of gestational weeks [1].

BPD was first coined by Northway et al. in 1967 [21]. Since then, subsequent studies gradually shed light on its pathophysiology. The pathology of BPD in a preterm neonate is as follows: pulmonary development in the neonate is inhibited after birth as the neonate with the disease starts pulmonary respiration while their lungs are underdeveloped. Since the structure of their lungs is not fully developed, the neonate suffers from hypoxemia, which may require mechanical ventilation management and the administration of oxygen. These interventions damage pulmonary tissue. Moreover, while cytokine-induced, intrauterine inflammatory changes frequently cause premature birth, a study has pointed out that inflammatory changes are also associated with pulmonary tissue injuries [22].

This study took into account the above-mentioned pathophysiological findings in analysing factors associated with the onset of BPD by using multivariate analysis. Existing studies have reported that oligohydramnios is a risk factor for BPD [12]. However, as mentioned above, it is considered that various factors are intricately interrelated when a neonate develops BPD, such as: premature birth, intrauterine infection and infection after birth, the sex of the neonate and SGA. After excluding the impact of confounding factors such as the number of gestational weeks and intrauterine infection, multivariate analysis in this study found that duration of oligohydramnios and preterm birth for less than 24.1 weeks are risk factors for BPD. Prolonged oligohydramnios may be associated with neonatal pulmonary hypoplasia [20, 23]. It has been pointed out that various factors, including premature lungs, oxygen toxicity and inflammatory mediators, are also involved [24]. Diagnostic criteria for and the definition of BPD vary, and consensus is yet to be reached [25, 26]. Meanwhile, risk factors for neonatal BPD during pregnancy include intrauterine growth restriction and chorioamnionitis [24]. One study reported that the incidence rate of BPD was higher in cases who experienced pPROM before 31 weeks of gestation than those who did not experience the condition [27], Another study reported that from among 36 neonates who were born to mothers diagnosed with pPROM before 24 weeks of gestation and who were discharged following delivery, 17 (47%) developed BPD [28]. Oligohydramnios was found to be a risk factor for BPD. It can be considered that neonates with BPD required the administration of oxygen after birth because the extensibility of their pulmonary tissue was reduced, from prolonged oligohydramnios that prevented the foetal development of the lungs.

It has been found that a neonate with BPD has increased mid- to long-term risks of pulmonary hypertension syndrome and chronic obstructive pulmonary disease [29]. Since this study did not examine the mid- to long-term prognosis of neonates, the studied neonates should be closely monitored for the onset of prolonged pulmonary hypertension going forward.

The ROC curve indicated that oligohydramnios that lasts four days or longer increases the risk of BPD. If a mother has persistent oligohydramnios for a period of four days or longer, an onset of BPD should be taken into account in managing the case. There has been some research that has reported that a neonate develops respiratory complications if prolonged oligohydramnios was observed in their mother (30). However, to the best of our knowledge, no research has been identified that explores the relationship between the duration of preterm oligohydramnios and the onset of neonatal BPD. The authors agree that the appropriateness of early pregnancy termination for a pPROM case should be determined on an individual basis by taking into account the number of gestational weeks and other individual background factors [18]. However, it is still necessary for health professionals to consider the risks of neonatal BPD when attempting to prolong pregnancy in a mother with persistent oligohydramnios in whom NRFS or clinical chorioamnionitis is not observed.

This study has several limitations. Firstly, this study is a retrospective epidemiological study which was performed at a single centre and did not involve large-scale data. Secondly, there may be a bias in selecting patients because the study site was a general perinatal care hospital which primarily accepted severe cases in its area. Finally, the endpoint for analysis was the onset of neonatal BPD. We were unable to follow-up the course of all postnatal babies because they were referred to other hospitals. Therefore, the presence of BPD complications was used as the short-term prognosis. Future studies may need to examine the long-term prognosis of neonates as well. Also, although the onset of BPD was examined by using an ROC curve, pregnancy termination was determined on an individual basis by taking into account the number of gestational weeks and patient background factors. Hence, it should be noted that interventions were performed at the discretion of the attending health professionals.

The following aspects should also be noted: firstly, this study is a retrospective epidemiological study which was performed at a single centre and which did not involve large-scale data; and secondly, there was a bias in selecting patients because the study site was a general perinatal care hospital which primarily accepts severe cases in its area.

In Table 3, there was no difference in the average value of gestational age at delivery (weeks) between the BPD group and non-BPD group, it was found that a significantly larger number of patients in the BPD group gave birth at gestational week 22–25. It was possible that the duration of oligohydramnios was significantly different between the two groups. In the BPD group, there were many preterm infants at gestational week 22–25, and the extension of gestational age has a great influence on the prognosis after birth for foetuses, so we sought to extend the gestational weeks even with oligohydramnios. In Table 3, there was no difference in the average gestational age at delivery (weeks) between the BPD group and non-BPD group, but there was a statistically significant difference in the number of infants delivered at gestational week 22–25. The significant difference in the duration of oligohydramnios between the 2 groups may have been because there were several extremely preterm babies (22–25 weeks) in the BPD group. The extension of gestational age has a great influence on the post-natal prognosis of foetuses at this gestational age. Therefore, we sought to extend the gestational age despite the presence of oligohydramnios. Although there was no statistical difference in the non-BPD group, many cases tended to approach late preterm. We believe this was partly because termination was actively considered.

## Conclusions

Findings from this study suggested that duration of oligohydramnios and gestational age at delivery for less than 24.1 weeks are risk factors for BPD in cases diagnosed with pPROM before 28 weeks of gestation. Also, the results of this study indicated that oligohydramnios that lasts four days or longer increases the risk of BPD. If pregnancy is extended and prolonged oligohydramnios is observed, the neonate should be closely monitored after birth for the onset of BPD.

## Data Availability

The datasets used and analysed during the current study are available from the corresponding author on reasonable request.

## Abbreviations

AUC: Area under the curve
BPD: Bronchopulmonary dysplasia
CAM: Chorioamnionitis
CI: Confidence interval
NRFS: Non-reassuring foetal status
ROC: Receiver operating characteristic
pPROM: Preterm premature rupture of membrane
SD: Standard deviation
SGA: Small for gestational age

## References

[1] Practice Bulletin No (2016). 172: Premature Rupture of Membranes. Obstet Gynecol.

[2] Niesluchowska-Hoxha A, Cnota W, Czuba B, Ruci A, Ciaciura-Jarno M, Jagielska A (2018). A Retrospective Study on the Risk of Respiratory Distress Syndrome in Singleton Pregnancies with Preterm Premature Rupture of Membranes between 24 + 0 and 36 + 6 Weeks, Using Regression Analysis for Various Factors. Biomed Res Int.

[3] Cutz E, Chiasson D (2008). Chronic lung disease after premature birth. N Engl J Med.

[4] Ericson JE, Laughon MM (2015). Chorioamnionitis: implications for the neonate. Clin Perinatol.

[5] Williams O, Hutchings G, Hubinont C, Debauche C, Greenough A (2012). Pulmonary effects of prolonged oligohydramnios following mid-trimester rupture of the membranes–antenatal and postnatal management. Neonatology.

[6] Blanc WA (1979). Pathology of the placenta and cord in ascending and in haematogenous infection. Ciba Found Symp..

[7] Jobe AH, Bancalari E (2001). Bronchopulmonary dysplasia. Am J Respir Crit Care Med.

[8] Ogunyemi D (2007). Risk factors for acute pulmonary edema in preterm delivery. Eur J Obstet Gynecol Reprod Biol.

[9] Lencki SG, Maciulla MB, Eglinton GS (1994). Maternal and umbilical cord serum interleukin levels in preterm labor with clinical chorioamnionitis. Am J Obstet Gynecol.

[10] Sandmire HF, DeMott RK (1998). Electronic fetal heart rate monitoring: research guidelines for interpretation. Am J Obstet Gynecol.

[11] Hanke K, Hartz A, Manz M, Bendiks M, Heitmann F, Orlikowsky T (2015). Preterm prelabor rupture of membranes and outcome of very-low-birth-weight infants in the German Neonatal Network. PLoS One.

[12] Weiner E, Barrett J, Zaltz A, Ram M, Aviram A, Kibel M (2019). Amniotic fluid volume at presentation with early preterm prelabor rupture of membranes and association with severe neonatal respiratory morbidity. Ultrasound Obstet Gynecol.

[13] Youden WJ (1950). Index for rating diagnostic tests. Cancer.

[14] Jaeschke R, Guyatt GH, Sackett DL (1994). Users’ guides to the medical literature. III. How to use an article about a diagnostic test. B. What are the results and will they help me in caring for my patients? The Evidence-Based Medicine Working Group. Jama.

[15] Bose C, Van Marter LJ, Laughon M, O’Shea TM, Allred EN, Karna P (2009). Fetal growth restriction and chronic lung disease among infants born before the 28th week of gestation. Pediatrics.

[16] Lemons JA, Bauer CR, Oh W, Korones SB, Papile LA, Stoll BJ (2001). Very low birth weight outcomes of the National Institute of Child health and human development neonatal research network, January 1995 through December 1996. NICHD Neonatal Research Network Pediatrics.

[17] Hartling L, Liang Y, Lacaze-Masmonteil T (2012). Chorioamnionitis as a risk factor for bronchopulmonary dysplasia: a systematic review and meta-analysis. Arch Dis Child Fetal Neonatal Ed.

[18] ACOG Obstetric Care Consensus No (2015). 3: Periviable Birth. Obstet Gynecol.

[19] Waters TP, Mercer BM (2009). The management of preterm premature rupture of the membranes near the limit of fetal viability. Am J Obstet Gynecol.

[20] Blott M, Greenough A (1988). Neonatal outcome after prolonged rupture of the membranes starting in the second trimester. Arch Dis Child.

[21] Northway WH, Rosan RC, Porter DY (1967). Pulmonary disease following respirator therapy of hyaline-membrane disease. Bronchopulmonary dysplasia. N Engl J Med.

[22] Jobe AJ (1999). The new BPD: an arrest of lung development. Pediatr Res.

[23] Hesson A, Langen E (2018). Outcomes in oligohydramnios: the role of etiology in predicting pulmonary morbidity/mortality. J Perinat Med.

[24] Kalikkot Thekkeveedu R, Guaman MC, Shivanna B (2017). Bronchopulmonary dysplasia: A review of pathogenesis and pathophysiology. Respir Med.

[25] Davidson LM, Berkelhamer SK (2017). Bronchopulmonary Dysplasia: Chronic Lung Disease of Infancy and Long-Term Pulmonary Outcomes. J Clin Med..

[26] Poindexter BB, Feng R, Schmidt B, Aschner JL, Ballard RA, Hamvas A (2015). Comparisons and Limitations of Current Definitions of Bronchopulmonary Dysplasia for the Prematurity and Respiratory Outcomes Program. Ann Am Thorac Soc.

[27] Pharande P, Mohamed AL, Bajuk B, Lui K, Bolisetty S (2017). Preterm infant outcomes in relation to the gestational age of onset and duration of prelabour rupture of membranes: a retrospective cohort study. BMJ Paediatr Open.

[28] McLaughlin LM, Gardener GJ (2016). Neonatal outcomes after prelabour rupture of membranes before 24 weeks’ gestation. J Paediatr Child Health.

[29] Wong PM, Lees AN, Louw J, Lee FY, French N, Gain K (2008). Emphysema in young adult survivors of moderate-to-severe bronchopulmonary dysplasia. Eur Respir J.

